# Synthesis, Anti-HCV, Antioxidant and Reduction of Intracellular Reactive Oxygen Species Generation of a Chlorogenic Acid Analogue with an Amide Bond Replacing the Ester Bond †

**DOI:** 10.3390/molecules21060737

**Published:** 2016-06-08

**Authors:** Ling-Na Wang, Wei Wang, Masao Hattori, Mohsen Daneshtalab, Chao-Mei Ma

**Affiliations:** 1 College of Life Sciences, Inner Mongolia University, Huhhot 010021, China; shxywln@163.com; 2 College of Biochemical Engineering, Huhhot Vocational College, Huhhot 010058, China; 3 Department of Chemistry, School of Pharmaceutical Sciences, Kunming Medical University, Kunming 650500, China; wangwei.kmu@hotmail.com; 4 Institute of Natural Medicine, University of Toyama, 2630 Sugitani, Toyama 930-0194, Japan; mhattori@po4.canet.ne.jp; 5 School of Pharmacy, Memorial University of Newfoundland, St. John’s, NL A1B 3V6, Canada

**Keywords:** 3-caffeoylquinic acid amide, synthesis, anti-HCV, anti-oxidant, cellular oxidative stress

## Abstract

Chlorogenic acid is a well known natural product with important bioactivities. It contains an ester bond formed between the COOH of caffeic acid and the 3-OH of quinic acid. We synthesized a chlorogenic acid analogue, 3α-caffeoylquinic acid amide, using caffeic and quinic acids as starting materials. The caffeoylquinc acid amide was found to be much more stable than chlorogenic acid and showed anti-Hepatitis C virus (anti-HCV) activity with a potency similar to chlorogenic acid. The caffeoylquinc acid amide potently protected HepG2 cells against oxidative stress induced by *tert*-butyl hydroperoxide.

## 1. Introduction

Chlorogenic acid is a natural product containing an ester bond formed between the COOH of caffeic acid and the 3-OH of quinic acid. Chlorogenic acid has been well known to have antioxidant and anti-viral activities, and the potential to be effective against type II diabetes, cardiovascular disease and some aging-related diseases [1,2,3,4]. Recent studies demonstrated that chlorogenic acid could attenuate d-galactolse-induced chronic liver and kidney injury, and the protection may be due to its antioxidative and anti-inflammatory effects [5]. Chlorogenic acid also showed therapeutic potential for inhibiting inflammatory hyperplasia of the synovium in patients with rheumatoid arthritis [6]. Using PC12 cells which are similar to neurons, researchers demonstrated that chlorogenic acid could increase the cell’s viability and differentiation by preventing the cells from alcohol-induced apoptosis [7]. Chlorogenic acid is used as a bioactive marker constituent for the quality control of some important traditional Chinese medicines (TCM), such as the flower of *Lonicera japonica* Thunb, the aerial parts of *Artemisia capillaries* Thunb and some frequently used TCM prescriptions containing these herb drugs [8].

Chlorogenic acid is also a useful starting material for the synthesis of derivatives with improved or new bioactivity. Adding a lipophilic chain at position 1 resulted in derivatives that could be taken up by hepatocytes and enhanced the inhibitory activity on hepatic glucose-6-phosphate translocase [9,10,11]. This enzyme catalyzes the final step in glycogenolytic and gluconeogenic pathways to liberate free glucose into the circulation, and inhibitors of this enzyme are potentially good for diabetic patients. Introducing a lipophilic chain and an amino acid residue to position 7 of chlorogenic acid led to potent anti-fungal compounds [12]. Adding lipophilic chains through acetal/ketal bonds to chlorogenic acid resulted in potent α–glucosidase inhibitors [13]. α-Glucosidase inhibitors could slow down the digestion and absorption of carbohydrates and thus are beneficial to type II diabetes patients.

**Scheme 1:**
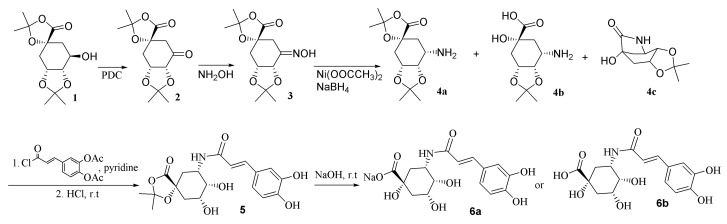
Synthesis of a chlorogenic acid analogue with an amide bond.

Because of the important roles of this compound in human health, scientists have been making much effort to find and improve the synthesis method of chlorogenic acid. However, as there are several hydroxyl groups, a carboxyl, and an ester group in the structure, the task is quite difficult. In 2001, Sefkow reported the synthesis in higher yield of a quinic acid bisacetonide (**1**, structure shown in Scheme 1), in which the 1-OH, 1-COOH and 4,5-OH were protected and only 3-OH was free, and efficiently synthesized chlorogenic acid by reacting **1** with acetylcaffeoyl chloride followed by de-protection [14].

It was observed that, although a small part of chlorogenic acid could be absorbed quickly, a large part of this compound was hydrolyzed *in vivo* through breakage of the ester bond between the caffeic and quinic acid moieties [15,16,17]. It is known that some bioactivity of chlorogenic acid could be lost if the ester bond was broken, such as in the case for the inhibition of hepatic glucose-6-phosphate translocase, chlorogenic acid showing good activity while neither caffeic acid nor quinic acid was active [9]. It is well documented that amides are more stable to esterase hydrolysis than esters, and if the ester bond of a compound was replaced by an amide bond, the stability would improve significantly [17,18]. Oxidative stress caused by reactive species of oxygen (ROS) damages cellular components and is recognized as one of the causes of chronic disease [19,20]. Human hepatoma cell line HepG2 is a reliable model for biochemical studies of intracellular antioxidant [21].

The present study aimed to synthesize and test the intracellular antioxidant activity of a chlorogenic acid analogue with an amide bond instead of the ester bond. In addition, the stability, anti-HCV activity, and toxicity on brine shrimps of this compound are described and compared with chlorogenic acid.

## 2. Results and Discussion

### 2.1. Synthesis of 5α-Caffeoylquinic Acid Amide

The first step was to synthesize 3-amino-3-deoxy-quinic acid. Due to the multiple hydroxyl groups in the structure of quinic acid, it is difficult to force the reaction to occur in the desired position. The authors of [14] solved this problem by synthesis of compound **1**. The present research investigated the method to convert the hydroxyl group to amino group as described in detail in the following passage. The desired product was obtained by acylation of the amino intermediate and finally de-protection. As both acid sensitive (4,5-ketal) and alkaline sensitive (7-ester) groups exist in the structure of **1**, reactions were carried out in conditions as mild as possible.

The synthesis route for the chlorogenic acid analogue from compound **1** [14] was depicted in Scheme 1. Compound **1** was firstly oxidized with pyridinium dichromate to give the ketone compound (**2**), which was then converted to the hydroxyimino compound (**3**) through reaction with NH_2_OH·HCl. The next step, reduction of compound **3** to amino compound, was found to be very difficult. Compound **3** could not be hydrogenated by H_2_-Ni or H_2_-Pt/C at 50 °C, neither could it react with Ni-2-propanol. TiCl_3_-NH_4_Ac changed **3** back to **2**, possibly through fast hydrolysis of an imine intermediate which was formed by reduction of **3**. Treatment of **3** with NaH_3_CN + TiCl_3_ resulted in a complex mixture, from which **4** could not be isolated. Finally, Ni(OOCCH_3_)_2_ and NaBH_4_ was used to react with **3** at low temperature. HR-MS revealed that the reaction mixture contained **4a**, **4b** and **4c**. The mixture was purified on an ODS column with the mobile phase kept at 0 °C to obtain a fraction containing mainly **4a** and **4b**. The mixture of **4a** and **4b** was acylated with acetylcaffeoyl chloride followed by de-protection to afford **6a** and **6b**.

Compound **6a** displayed m/z at 352 in negative ESI-MS. High resolution-FAB-MS [M − H]^−^ showed *m*/*z* 352.10167 (Calcd. For C_16_H_18_O_8_N, requires 352.10322). Its ^1^H-NMR is quite similar to that of chlorogenic acid except that the H-5 signals at δ 4.56 in **6a** (δ 4.23 in **6b**) is up-field shifted compared to that of chlorogenic acid at δ 5.33. These data indicated that the ester bond was converted to an amide bond.

Compounds **6a** and **6b** were confirmed to be the salt form and free form of the same compound based on the following evidences. The mixture of **6a** and **6b** gave only one peak in the LC-MS analysis using an acidic mobile phase containing 0.1% HCOOH. When **6a** was acidified with TFA and then concentrated, its ^1^H-NMR spectrum became exactly the same as that of **6b**.

**Figure 1:**
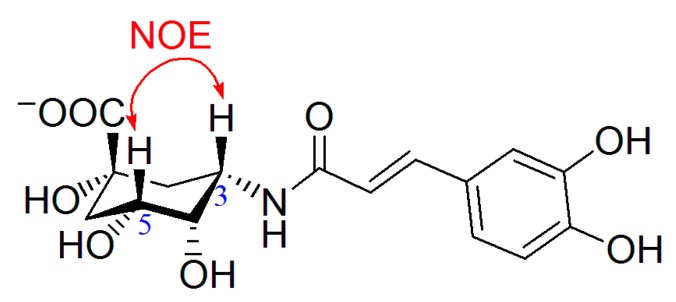
NOE interaction defining the configuration of C-3 in **6a**.

To determine the orientation of the amide bond, nuclear Overhauser enhancement (NOE) experiment (see supporting material) was carried out for **6a**. When H-3 was irradiated, significant NOE effects were observed for H-2, H-4 and H-5, therefore H-3 was in the same orientation as H-5, *i.e.*, in β-form, and thus the 3-amide bond is in α-orientation (Figure 1). For comparison, the NOE experiment of chlorogenic acid was also carried out (see supporting material). The H-3 in chlorogenic acid was in α-form and no NOE effect could be observed for H-5 when H-3 was irradiated.

### 2.2. Antioxidant Activity

Using reported methods [21], the anti-oxidant activities of **6b** and chlorogenic acid were compared. The results were expressed as EC_50_ representing the concentration (μg/mL) that produced 50% reduction of 2,2-diphenyl-1-picrylhydrazyl (DPPH) and IC_50_ representing the concentration (μg/mL) of SOD-like compound that inhibited the formation of WST-1 formazan by 50%. The EC_50_ and IC_50_ values for **6b** were found to be 17 (μg/mL) and 1.6 (μg/mL), respectively, and those for chlorogenic acid were 17 (μg/mL) and 1.5 (μg/mL), respectively. The results indicated that the amide analogue retained the strong anti-oxidant activity of chlorogenic acid.

### 2.3. Protection on HepG2 against t-BuOOH Induced Oxidative Stress

**Figure 2:**
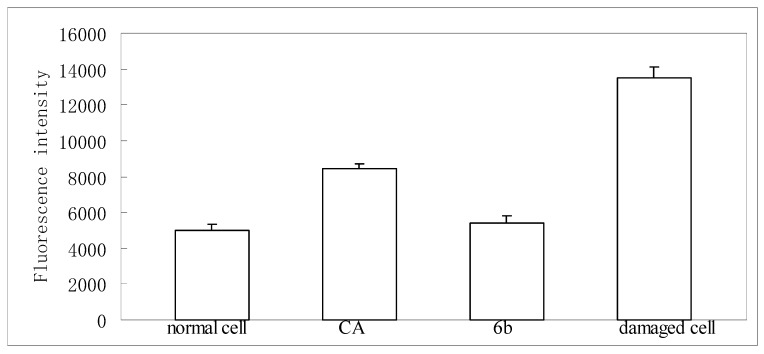
Inhibitory effect of chlorogenic acid (CA) and its amide analogue (**6b**) on reactive species of oxygen (ROS) formation.

Cytotoxic assay indicated that neither chlorogenic acid nor compound **6** were toxic on HepG-2 at 50 μg/mL. In a dichlorofluorescein assay [18], a significant increase of ROS generation was observed in HepG2 cells treated with *t*-BuOOH as compared to non-stressed controls. Pretreatment with chlorogenic acid or compound **6** at 50 μmol/L remarkably decreased ROS generation as expressed by fluorescence (Figure 2).

### 2.4. Anti-HCV Activity

Anti-HCV activity was assessed by measuring the EGFP autofluorescence in HCV infected Huh-7.5 cell lines [22,23]. The values represent a mean of triplicate results of the declined percentage of EGFP autofluorescence activity. Compound **6b** and chlorogenic acid showed 49.5% and 58.5% of inhibition, respectively, at a concentration of 100 μg/mL. The results indicated that both chlorogenic acid and its amide analogue possess anti-HCV activity.

### 2.5. Toxicity on Brine Shrimps

The toxicity of chlorogenic acid and compound **6** was evaluated with *Artemia salina* L. (brine shrimps) assay using a reported method [24]. The concentration that caused 50% mortality (LC_50_) of brine shrimp by chlorogenic acid and compound **6b** were found to be 300 and 500 μg/mL, respectively. The decreased toxicity of compound **6b** as compared with chlorogenic acid may come from the much lower propensity for **6b** to decompose forming more toxic compound, caffeic acid.

### 2.6. Stability of Chlorogenic Acid and 5α-Caffeoylquinic Acid Amide

Stability tests were carried out in phosphate buffer (PBS) (pH 7.4) containing MgCl_2_, rat liver microsomes and β-NADPH. After 15 h incubation, about 50% of chlorogenic acid was decomposed to caffeic acid and quinic acid, while compound **6b** was intact without any caffeic acid and quinic acid detected. This result is in accordance with the general metabolic concept that amides are more stable than esters [18].

## 3. Experimental Section

### 3.1. Materials and Apparatus

Caffeic acid, quinic acid and other chemical reagents were purchased from Sigma-Aldrich Corporation (St. Louis, MO, USA). Analytical grade solvents were used in the whole process. High performance liquid chromatography (HPLC) grade solvents used for UPLC were purchased from Fisher Scientific Company (Fair Lawn, NJ, USA). Octadecylsilane (ODS, 38–63 μm) was purchased from Wako Pure Chemical Industries, Ltd., Osaka, Japan, Sephadex LH-20 was purchased from GE Healthcare Bio-Sciences AB, Uppsala, Sweden. NMR spectra were measured with a Varian Unity 500 (^1^H, 500 MHz; ^13^C, 125 MHz) spectrometer (Varian Co., Palo Alto, CA, USA) or a Bruker-500 (^1^H, 500 MHz; ^13^C, 125 MHz) NMR spectrometer (Bruker Inc., Fällanden, Switzerland). UPLC-DAD-ESI-MS experiments were carried out on an Agilent 1290 infinity UPLC-DAD system (Agilent Technologies Singapore (International) Pte. Ltd., Singapore) coupled with an Agilent 6340 triple quad MS. Optical rotations were measured on a JASCO DIP-360 automatic polarimeter (JASCO Co., Tokyo, Japan). High resolution mass spectrum was measured on a Xevo G2 Q-TOF mass spectrometer (Waters, Milford, MA, USA) or a JEOL JMS-AX505W FAB-MS spectrometer (JEOL Co., Tokyo, Japan) with a resolution of 5000 using m-nitrobenzyl alcohol as the matrix. NMR spectra and HRMS data associated with this article can be found in the Supplementary.

### 3.2. Chemical Synthesis

The synthesis route was depicted in Scheme 1. Compound **1** [14] (23.2 g, 85.2 mmol), pyridinium dichromate (50 g, 231.9 mmol) and molecular sieves 3 Ǻ (70 g) were stirred in CH_2_Cl_2_ (300 mL) at r.t. overnight. The mixture was passed through a short SiO_2_ column eluted with AcOEt:MeOH 99:1–95:5 to get **2** (22.1 g, 81.8 mmol) in 96% yield. To a pyridine solution (100 mL) of **2** (18 g, 66.6 mmol) was added NH_2_OH·HCl (16.3 g, 228.0 mmol). The mixture was stirred at 50 °C for 3 h followed by addition of 50 times (in volume) of 0.137 M aq. HCl. The mixture was passed through an ODS column eluted with MeOH-H_2_O. The product (**3**, 14 g, 49.1 mmol, 73.7%) was obtained from the 20% to 40% MeOH eluted part. To **3** (0.7 g, 2.5 mmol) in 7 mL MeOH was added Ni(OOCCH_3_)_2_ (1.4 g, 7.91 mmol). The mixture was cooled in ice followed by addition of NaBH_4_ (0.65 g, 17.2 mmol) powder. The mixture was stirred at 0 °C for 4 h followed by addition of 30 mL ice-H_2_O. The mixture was purified on ODS column while keeping the mobile phase at 0 °C to obtain a fraction containing mainly **4a** and **4b** (250 mg) from 20% to 50% MeOH eluted part. To a CH_2_Cl_2_ solution (10 mL) of **4a** and **4b** (200 mg) were added DMAP (32 mg, 0.30 mmol), pyridine (3 mL) and acetylcaffeoyl chloride (0.56 g, 2.0 mmol). The reaction mixture was stirred at r.t. for 5 h and acidified with 1 M aq. HCl to pH = 3. The organic solvent was evaporated and the residue was passed through an ODS column. The 60%–100% methanol eluted part was evaporated to dryness followed by treatment with 10 mL MeOH-H_2_O (4:1) solution of 0.5 M HCl at r.t. overnight. After being concentrated to dryness, the mixture was treated with 6 mL 1 M NaOH and 9 mL MeOH at r.t. for 1 h and then neutralized with 1 M HCl to pH = 6. The organic solvent was evaporated *in vacuo* at 40 °C and the residue passed through an ODS column eluted with H_2_O and then 90% MeOH. The 90% MeOH eluted part was concentrated and further purified on a Sephadex LH 20 column eluted with H_2_O containing increasing amount of MeOH. Compound **6a** was obtained from the 10% MeOH eluted part (115 mg).

Acylation of **4a** and **4b** (100 mg) was carried in the same manner and deprotection of the intermediate product in the same procedure as described above, except for adjusting the solution to pH = 3 in the last step, to obtain 50 mg of **6b**.

### 3.3. Spectral Data

Compound **2**: white solid. HR-ESI-MS [M + Na]^+^ *m*/*z* 293.1004 (Calcd. For C_13_H_18_O_6_Na, requires 293.1001).

Compound **3**: grey solid. HR-ESI-MS [M + H]^+^ *m*/*z* 286.1298 (Calcd. For C_13_H_20_NO_6_, requires 286.1291).

Compound **4**: grey solid. HR-ESI-MS of **4a** [M + H]^+^ *m*/*z* 272.1502 (Calcd. For C_13_H_22_NO_5_, requires 272.1498); HR-ESI-MS of **4b** [M + H]^+^ *m*/*z* 232.1190 (Calcd. For C_10_H_18_NO_5_, requires 232.1185), [M − H]^−^ *m*/*z* 230.1034 (Calcd. For C_10_H_16_NO_5_, requires 230.1028); HR-ESI-MS of **4c** [M + H]^+^ *m*/*z* 214.1082 (Calcd. For C_10_H_16_NO_4_, requires 214.1079).

Compound **5**: grey solid. HR-ESI-MS [M + H]^+^ *m*/*z* 394.1508 (Calcd. For C_19_H_24_NO_8_, requires 394.1502), [M + Na]^+^ *m*/*z* 416.1327 (Calcd. For C_19_H_23_NO_8_Na, requires 416.1321), [M − H]^−^ *m*/*z* 392.1343 (Calcd. For C_19_H_22_NO_8_, requires 392.1345).

Compound **6a** (Sodium salt of (1*R*,3*S*,4*S*,5*R*)-3α-[3-(3,4-dihydroxy-phenyl)-acryloylamino]-1,4,5-trihydroxy-cyclohexanecarboxylic acid): white solid. ${\left[\alpha \right]}_{D}^{24}$ −14.8 (*c* 0.19, CH_3_OH). ^1^H-NMR (CD_3_OD, 500 MHz), δ 1.87 (m, 4H,H-2a, 2b and 6a, 6b), 3.94 (t, *J* = 2.5 Hz, 1H, H-4), 4.25 (ddd, *J* = 2.5, 4.5, 10.5 Hz,1H, H-5), 4.56 (ddd, *J* = 2.5, 5.0, 10.5 Hz, 1H, H-3), 6.43 (d, *J* = 15.5 Hz, 1H, H-8′), 6.75 (d, *J* = 8.0 Hz, 1H, H-5′), 6.89 (dd, *J* = 2.0, 8.0 Hz, 1H, H-6′), 7.00 (d, *J* = 2.0 Hz, 1H, H-2′), 7.37 (d, *J* = 15.5 Hz, 1H, H-7′). ^13^C-NMR (CD_3_OD, 125 MHz), δ 36.9 (C-2), 39.2 (C-6), 49.7 (C-3), 69.9 (C-5), 71.6 (C-4), 74.6 (C-1), 115.0 (C-2′), 116.4 (C-5′), 118.9 (C-8′), 122.1 (C-6′), 128.4 (C-1′), 142.0 (C-7′),146.7 (C-4′), 148.7 (C-3′), 168.8 (C-9′), 181.9 (C-7, salt form).

Compound **6b** (Free acid of (1*R*,3*S*,4*S*,5*R*)-3α-[3-(3,4-Dihydroxy-phenyl)-acryloylamino]-1,4,5-trihydroxy-cyclohexanecarboxylic acid): white solid. ${\left[\alpha \right]}_{D}^{24}$ −26.1 (*c* 0.5, CH_3_OH). ^1^H-NMR (CD_3_OD, 500 MHz), δ 1.90 (m, 2H,H-2a, H-6a), 2.02 (ddd, *J* = 3.0, 4.5, 13.0 Hz, 1H, H-2b), 2.14 (dd, *J* = 4.0, 13.0 Hz, 1H, H-6b), 3.91 (m, H-4, 5), 4.23 (ddd, *J* = 2.0, 4.5, 12.5 Hz, 1H, H-3), 6.46 (d, *J* = 15.5 Hz, 1H, H-8′), 6.77 (d, *J* = 8.0 Hz, 1H, H-5′), 6.92 (dd, *J* = 2.0, 8.0 Hz, 1H, H-6′), 7.03 (d, *J* = 2.0 Hz, 1H, H-2′), 7.40 (d, *J* = 15.5 Hz, 1H, H-7′). ^13^C-NMR (CD_3_OD, 125 MHz), δ 35.8 (C-2), 38.1 (C-6), 49.3 (C-3), 69.1 (C-5), 71.2 (C-4), 73.9 (C-1), 115.1 (C-2′), 116.4 (C-5′), 118.5 (C-8′), 122.1 (C-6′), 128.4 (C-1′), 142.4 (C-7′),146.7 (C-4′), 148.7 (C-3′), 168.4 (C-9′), 177.4 (C-7). ESI-MS (Negative): *m*/*z* 352.0 [M − H]^−^; HR-FAB-MS [M − H]^−^ *m*/*z* 352.10167 (Calcd. For C_16_H_18_O_8_N, requires 352.10322).

NOE experiment of **6a**: When H-3 at δ 4.56 was irradiated, significant NOE effects were observed for H-4, H-5 and H-2 at δ 3.94, 4.25 and 1.87, respectively.

NOE experiment of chlorogenic acid: When H-3 at δ 5.33 was irradiated, significant NOE effects were observed for H-4 and H-2 at δ 3.72 and 2.21, respectively, but no NOE effect for H-5 at δ 4.17.

### 3.4. Measurement of Radical Scavenging Activity and Superoxide Dismutase (SOD)-Like Activity

Radical scavenging activity was evaluated on 96-well plates. To each well was added 10 μL DMSO solution of a compound and 190 μL ethanol solution of DPPH (0.1 mM). The mixture was kept at rt for 20 min before the absorbance at 540 nm was measured using a plate reader (DNM-9602, Beijing Pu Long new technology Co. Ltd., Beijing, China). The radical scavenging activity was calculated as (effective rate%) = 100 × (A_control_ − A_compound_)/A_control_, where control wells contained DMSO instead of compound solution. Compounds were tested in triplicate at 4 concentrations and the EC_50_ values were calculated from the curves of effective rate% *vs* compound concentrations.

SOD-like activity was evaluated on 96-well plates using an SOD Assay Kit-WST (Dojindo Chemical, Kumamoto, Japan). To each well was added 20 μL sample solution, 200 μL WST working solution and 20 μL xanthine oxidase solution. The plates were incubated at 37 °C for 20 min before the absorbance at 450 nm was measured with the plate reader. The SOD-like activity represented as inhibition rate% was calculated with the following equation. SOD-like activity (inhibition rate%) = 100 × ((A_blank_1 − A_blank_3) − (A_sample_ − A_blank_2))/(A_blank_1 − A_blank_3). Blank 1 contained water instead of sample solution; blank 2 contained buffer instead of enzyme; blank 3 contained water and buffer instead of sample solution and enzyme.

### 3.5. Test for Anti-HCV Activity

Anti-HCV activities were evaluated *in vitro* in an authentic HCV infection system in human hepatoma cell lines by measuring the EGFP autofluorescence as described in the literature [22,23]. Compound **6b** and chlorogenic acid were tested at a final concentration of 100 μg/mL. A positive control (HCV-796) [22] was tested at 0.4 μM (showed 46.6% of inhibition).

### 3.6. Assay of the Toxicity on Brine Shrimps

Brine shrimp assay was carried out using reported methods [24,25] with some modification. Ten larvae were placed in a tube containing 3.8% NaCl water solution and different concentrations of compounds. After 2 h, the survivor larvae were counted and the concentration that caused 50% mortality (LC_50_) of brine shrimp was calculated.

### 3.7. Cell Culture and Cytotoxic Assay

HepG-2 cells in DMEM medium supplemented with 10% FBS and 50 units/mL penicillin and 50 μg/mL streptomycin were cultured in a 5% CO_2_ atmosphere at 37 °C. Cells were seeded in 96-well plates at a rate of 50,000 cells per well. After addition of **6b** and chlorogenic acid, the cells were cultured for 48 h. MTT in PBS (20 μL of 5 mg/mL) was added to each well and the plates were incubated at 37 °C. Four hours later, the medium was aspirated and the formazan was dissolved in DMSO for determination of absorbance at 570 nm.

### 3.8. Determination of ROS Formation in HepG2 Cells

Determination of ROS formation in HepG2 cell was carried out using the dichlorofluorescin (DCFH) assay [26]. DCFH is non-fluorescent and can across the cell membrane freely. Intracellular ROS can oxidize DCFH to the fluorescent dichlorofluorescein DCF and the overall ROS could be estimated by measuring the fluorescence.

HepG-2 cells were plated in 96-well plate at a density of 20,000 cells per well and incubated for 4 h before chlorogenic acid and compound **6b** were added and further incubated for 24 h. After the cells were washed once with a serum-free medium, 100 μL of 10 μM DCFH was added to each well. The plates were further incubated at 37 °C for 30 min. The cells were washed twice with a serum-free medium and then treated with 100 μL of 400 μM t-BuOOH and incubated for 1.5 h. The liquid was discarded and 100 μL PBS was added to each well, and the plates were immediately measured with a Tecan Infinite F200 PRO microplate reader at excitation/emission wavelengths of 490 nm/520 nm. The results were represented as the fluorescence intensity.

### 3.9. Stability Test

Stability test was carried out in phosphate buffered (PBS) (pH 7.4) containing 5.0 mM of MgCl_2_. The reaction mixture comprised of 5 μL DMSO solution of tested compound (10 mg/mL) and 100 μL rat liver microsomes (protein 1.0 mg). The reaction was triggered with 5 μL of β-NADPH (2.0 mM) and incubated at 37 °C under agitation. An aliquot of 100 μL of the mixture was taken at different time interval, and the reaction was stopped by addition of 200 μL ice-cooled ethyl acetate. The mixture was evaporated *in vacuo* and the residue was dissolved in 80 μL of methanol for LC-MS analysis.

## 4. Conclusions

We synthesized the chlorogenic acid analogue, 3α-caffeoylquinic acid amide. This chlorogenic acid analogue was more stable than chlorogenic acid and retained the antioxidant and anti-viral activities of chlorogenic acid. The chlorogenic acid analogue showed stronger protective activity than chlorogenic acid on HepG-2 cells from tert-butyl hydroperoxide induced oxidation.

Chlorogenic acid has been used as a starting material to synthesize promising drug candidates. Using the presently synthesized amide analogue of chlorogenic acid as a starting material, it will be possible to synthesize more stable derivatives. The present procedure could also be applied to synthesize other acyl quinic acid amides, such as *p*-coumaroyl, feruloyl or galloylquinic acid amides. All the ester counterparts of these compounds are important bioactive natural products.

## Supplementary Materials

The NMR and MS spectra are available online at www.mdpi.com/1420-3049/21/6/737/s1.
