# Supplementary material for: Synthesis, Anti-HCV, Antioxidant and Reduction of Intracellular Reactive Oxygen Species Generation of a Chlorogenic Acid Analogue with an Amide Bond Replacing the Ester Bond
Source: Molecules. 2016 Jun 8;21(6):737. doi: 10.3390/molecules21060737 (PMC6273953; doi:10.3390/molecules21060737)
Supplement: Supplementary file 1 [file molecules-21-00737-s001.pdf]

Supplementary Materials: Synthesis, Anti-HCV, Antioxidant and Reduction of Intracellular Reactive Oxygen Species Generation of a Chlorogenic Acid Analogue with an Amide Bond Replacing the Ester Bond

Ling-Na Wang, Wei Wang, Masao Hattori, Mohsen Daneshtalab and Chao-Mei Ma

(+) HRESIMS of compound **2** S2
(+) HRESIMS of compound **3** S3
(+) HRESIMS of compound **4** S4
(–) HRESIMS of **4** S6
(+) HRESIMS of **5** S7
(–) HRESIMS of **5** S8
NOE spectrum of **6a** S9
NOE spectrum of chlorogenic acid S9
^{1}H-NMR spectrum of **6a** S10
^{13}C-NMR spectrum of **6a** S10
^{1}H-NMR spectrum of **6b** S11
^{13}C-NMR spectrum of **6b** S11
HRFABMS(-) spectrum of **6a** S12

(+) HRESIMS of compound **2**

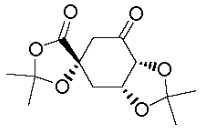

2 $C_{13}H_{18}O_6$
Exact Mass: 270.1103

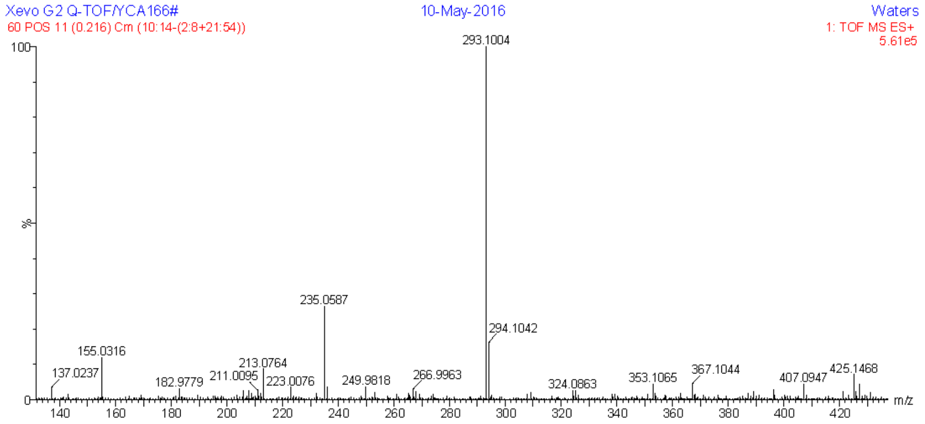

Elemental Composition Report of [**2+Na**]$^+$

Single Mass Analysis

Tolerance = 5.0 PPM / DBE: min = -1.5, max = 50.0

Element prediction: Off

Number of isotope peaks used for i-FIT = 3

Monoisotopic Mass, Even Electron Ions

508 formula(e) evaluated with 2 results within limits (up to 50 closest results for each mass)

Elements Used: C: 0-100 H: 0-200 N: 0-5 O: 0-20 Na: 0-1

Minimum: -1.5

Maximum: 5.0 5.0 50.0

Mass	Calc. Mass	mDa	PPM	DBE	i-FIT	Norm	Conf(%)	Formula
293.1004	293.1001	0.3	1.0	4.5	223.4	0.386	67.95	C13 H18 O6 Na

(+) HRESIMS of compound **3**

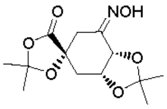

3 $C_{13}H_{19}NO_6$
Exact Mass: 285.1212

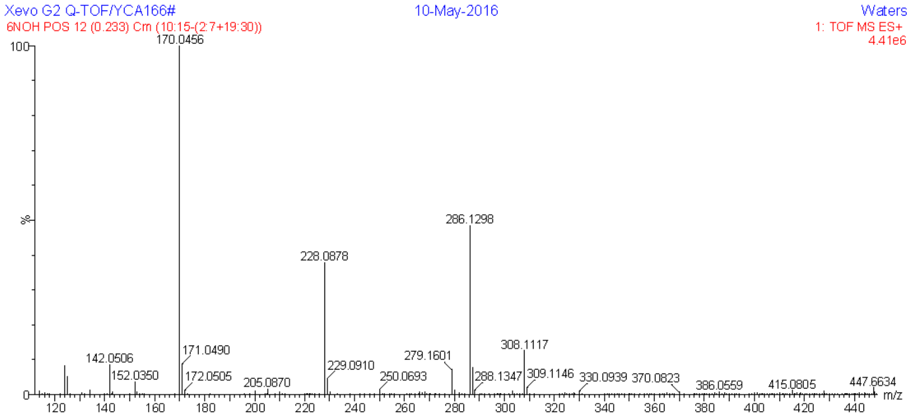

Elemental Composition Report of [**3+H**]$^+$

Single Mass Analysis

Tolerance = 5.0 PPM / DBE: min = -1.5, max = 50.0

Element prediction: Off

Number of isotope peaks used for i-FIT = 3

Monoisotopic Mass, Even Electron Ions

482 formula(e) evaluated with 2 results within limits (up to 50 closest results for each mass)

Elements Used:C: 0-100 H: 0-200 N: 0-5 O: 0-20 Na: 0-1

Minimum:					-1.5				
Maximum:			5.0	5.0	50.0				
Mass		Calc. Mass	mDa	PPM	DBE	i-FIT	Norm	Conf(%)	Formula
286.1298		286.1291	0.7	2.4	4.5	342.1	0.008	99.19	C13 H20 N O6

(+) HRESIMS of compound **4**

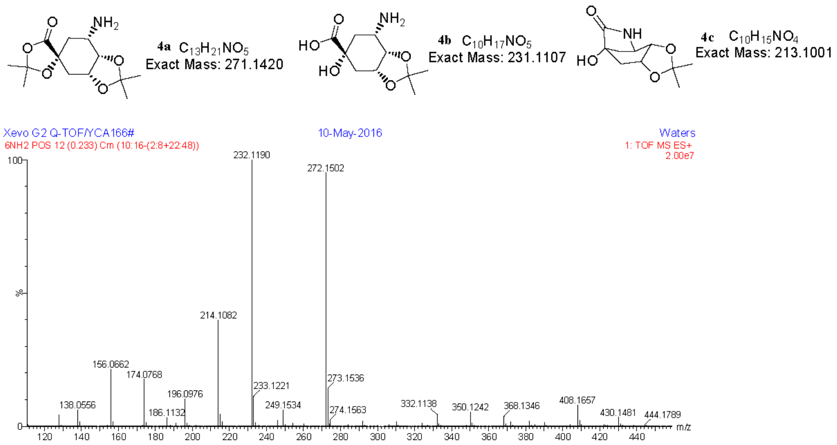

Elemental Composition Report of [**4a+H**]+

Single Mass Analysis

Tolerance = 5.0 PPM / DBE: min = -1.5, max = 50.0

Element prediction: Off

Number of isotope peaks used for i-FIT = 3

Monoisotopic Mass, Even Electron Ions

439 formula(e) evaluated with 2 results within limits (up to 50 closest results for each mass)

Elements Used: C: 0-100 H: 0-200 N: 0-5 O: 0-20 Na: 0-1

Mass	Calc. Mass	mDa	PPM	DBE	i-FIT	Norm	Conf(%)	Formula
Minimum:				-1.5				
Maximum:		5.0	5.0	50.0				
272.1502	272.1498	0.4	1.5	3.5	659.7	0.003	99.71	C13 H22 N O5

Elemental Composition Report of [**4b+H**]+

Single Mass Analysis

Tolerance = 5.0 PPM / DBE: min = -1.5, max = 50.0

Element prediction: Off

Number of isotope peaks used for i-FIT = 3

Monoisotopic Mass, Even Electron Ions

332 formula(e) evaluated with 2 results within limits (up to 50 closest results for each mass)

Elements Used: C: 0-100 H: 0-200 N: 0-5 O: 0-20 Na: 0-1

Mass	Calc. Mass	mDa	PPM	DBE	i-FIT	Norm	Conf(%)	Formula
Minimum:				-1.5				
Maximum:		5.0	5.0	50.0				
232.1190	232.1185	0.5	2.2	2.5	594.8	0.002	99.83	C10 H18 N O5

Elemental Composition Report of [**4c+H**]+

Single Mass Analysis

Tolerance = 5.0 PPM / DBE: min = -1.5, max = 50.0

Element prediction: Off

Number of isotope peaks used for i-FIT = 3

Monoisotopic Mass, Even Electron Ions

286 formula(e) evaluated with 2 results within limits (up to 50 closest results for each mass)

Elements Used:C: 0-100 H: 0-200 N: 0-5 O: 0-20 Na: 0-1

Minimum:				-1.5				
Maximum:		5.0	5.0	50.0				
Mass	Calc. Mass	mDa	PPM	DBE	i-FIT	Norm	Conf(%)	Formula
214.1082	214.1079	0.3	1.4	3.5	577.3	0.322	72.47	C10 H16 N O4

(–) HRESIMS of **4**

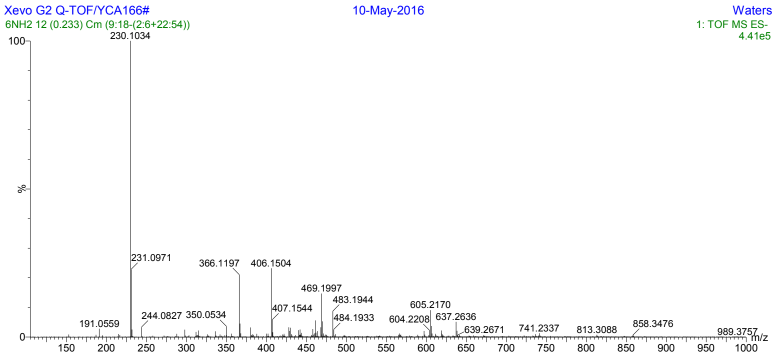

Elemental Composition Report of [**4b-H**]⁻

Single Mass Analysis

Tolerance = 5.0 PPM / DBE: min = -1.5, max = 50.0

Element prediction: Off

Number of isotope peaks used for i-FIT = 3

Monoisotopic Mass, Even Electron Ions

327 formula(e) evaluated with 2 results within limits (up to 50 closest results for each mass)

Elements Used:C: 0-100 H: 0-200 N: 0-5 O: 0-20 Na: 0-1

Minimum:				-1.5				
Maximum:		5.0	5.0	50.0				
Mass	Calc. Mass	mDa	PPM	DBE	i-FIT	Norm	Conf(%)	Formula
230.1034	230.1028	0.6	2.6	3.5	190.3	1.160	31.36	C10 H16 N O5

(+) HRESIMS of **5**

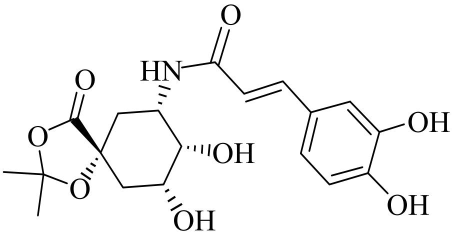

5 $C_{19}H_{23}NO_8$
Exact Mass: 393.1424

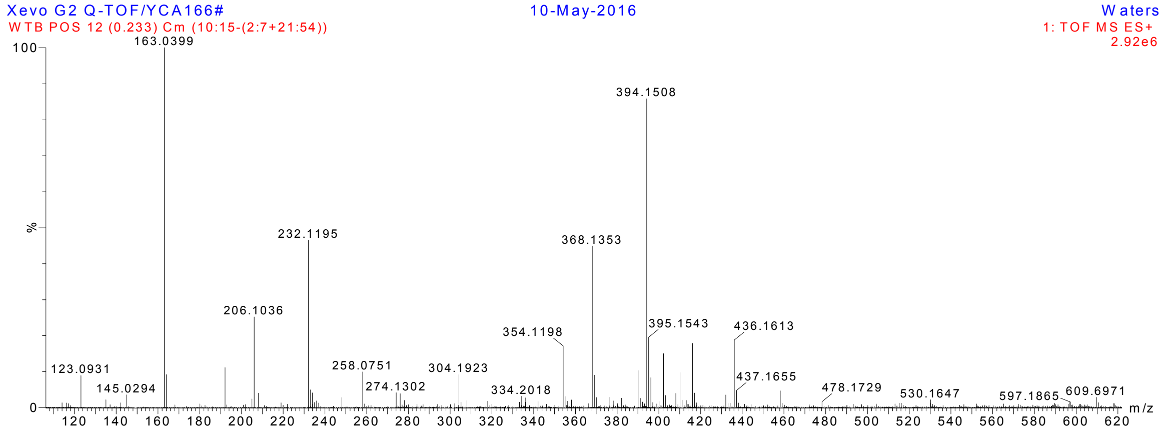

Elemental Composition Report of [**5+H**]+

Single Mass Analysis

Tolerance = 5.0 PPM / DBE: min = -1.5, max = 50.0

Element prediction: Off

Number of isotope peaks used for i-FIT = 3

Monoisotopic Mass, Even Electron Ions

846 formula(e) evaluated with 3 results within limits (up to 50 closest results for each mass)

Elements Used:C: 0-100 H: 0-200 N: 0-5 O: 0-20 Na: 0-1

Minimum:				-1.5				
Maximum:		5.0	5.0	50.0				
Mass	Calc. Mass	mDa	PPM	DBE	i-FIT	Norm	Conf(%)	Formula
394.1508	394.1502	0.6	1.5	8.5	379.8	0.867	42.01	C19 H24 N O8

Elemental Composition Report of [**5+Na**]+

Single Mass Analysis

Tolerance = 5.0 PPM / DBE: min = -1.5, max = 50.0

Element prediction: Off

Number of isotope peaks used for i-FIT = 3

Monoisotopic Mass, Even Electron Ions

915 formula(e) evaluated with 3 results within limits (up to 50 closest results for each mass)

Elements Used: C: 0-100 H: 0-200 N: 0-5 O: 0-20 Na: 0-1

Minimum:				-1.5				
Maximum:		5.0	5.0	50.0				
Mass	Calc. Mass	mDa	PPM	DBE	i-FIT	Norm	Conf(%)	Formula
416.1327	416.1321	0.6	1.4	8.5	329.3	1.330	26.46	C19 H23 N O8 Na

(–) HRESIMS of **5**

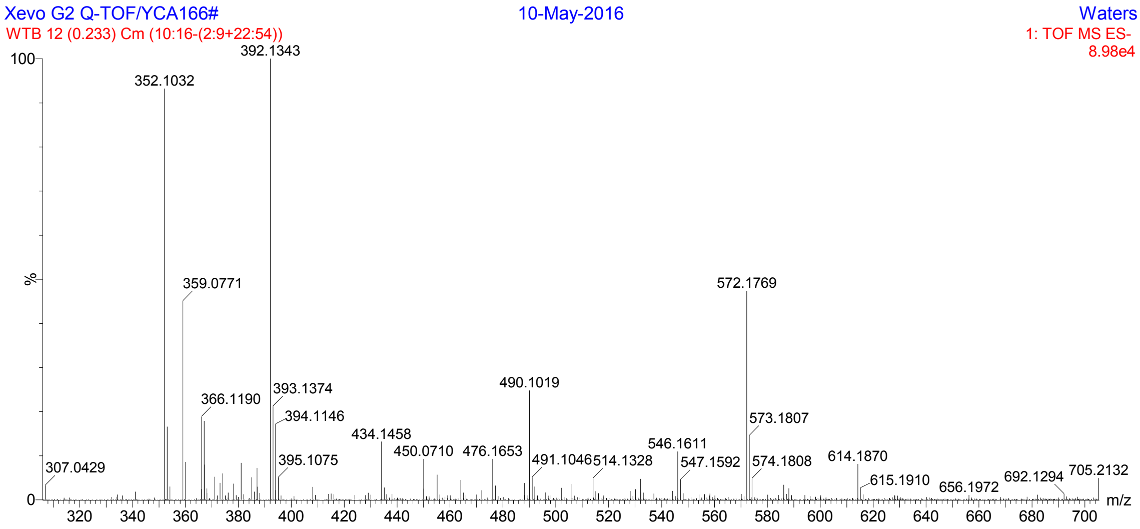

Elemental Composition Report of [**5-H**]$^-$

Single Mass Analysis

Tolerance = 5.0 PPM / DBE: min = -1.5, max = 50.0

Element prediction: Off

Number of isotope peaks used for i-FIT = 3

Monoisotopic Mass, Even Electron Ions

840 formula(e) evaluated with 3 results within limits (up to 50 closest results for each mass)

Elements Used: C: 0-100 H: 0-200 N: 0-5 O: 0-20 Na: 0-1

Mass	Calc. Mass	mDa	PPM	DBE	i-FIT	Norm	Conf(%)	Formula
Minimum:				-1.5				
Maximum:		5.0	5.0	50.0				
392.1343	392.1345	-0.2	-0.5	9.5	154.1	2.425	8.85	C19 H22 N O8

NOE spectrum of **6a**

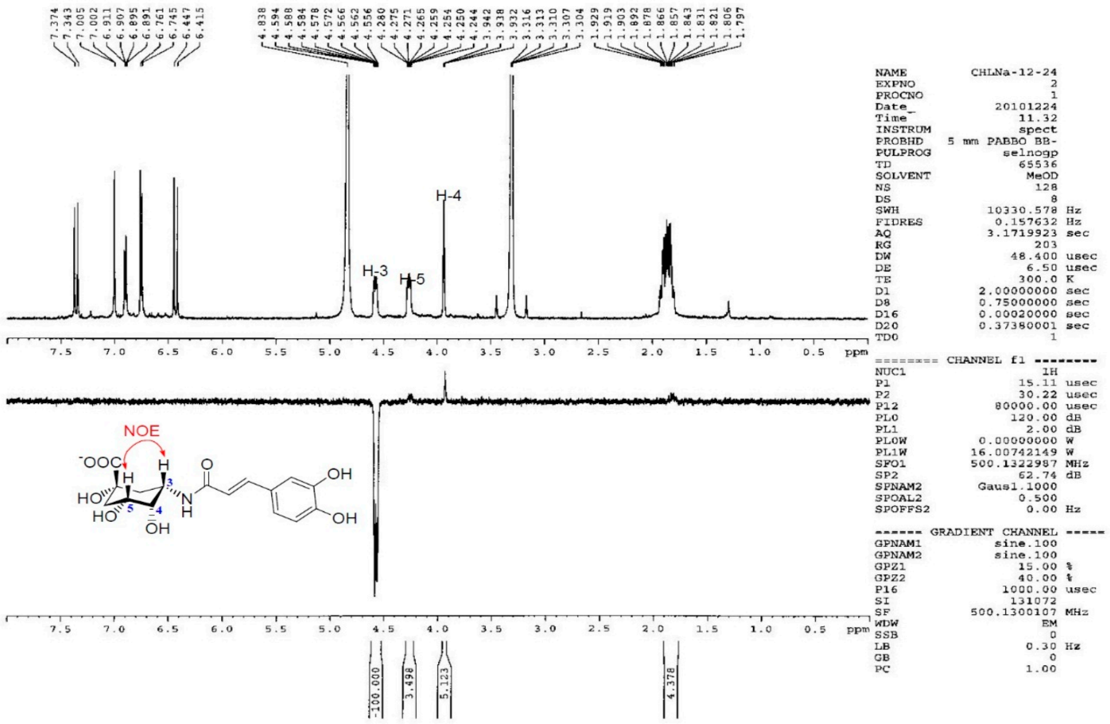

NOE spectrum of chlorogenic acid

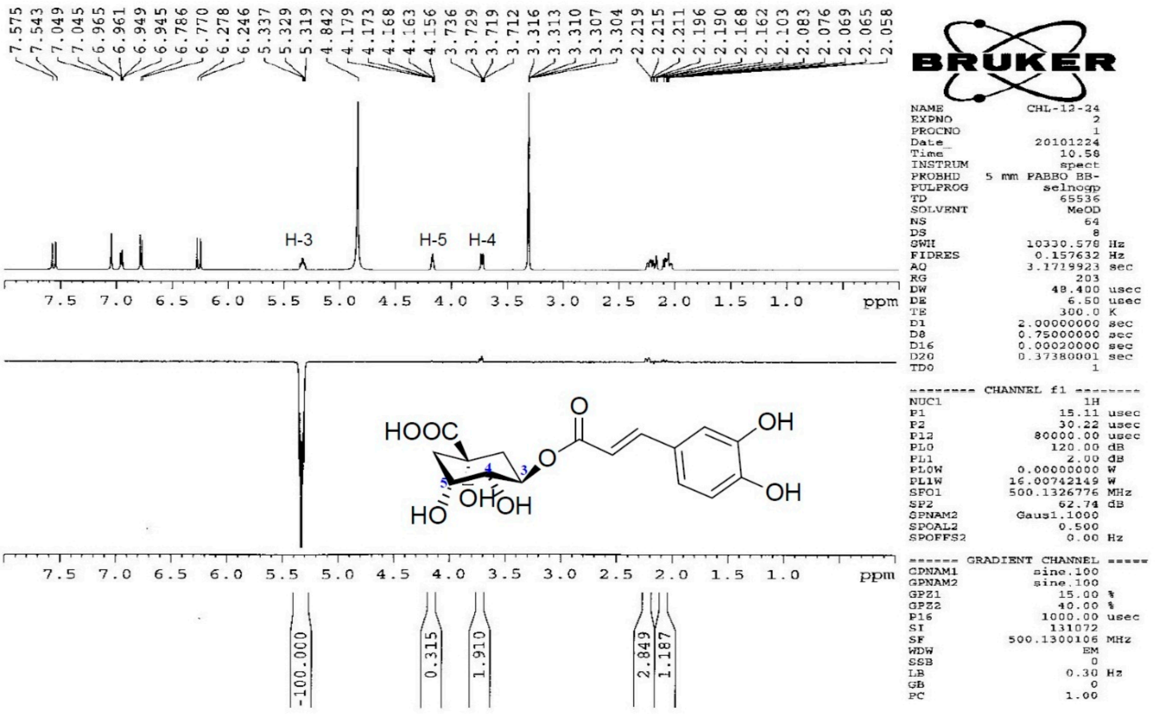

^{1}H-NMR spectrum of **6a**

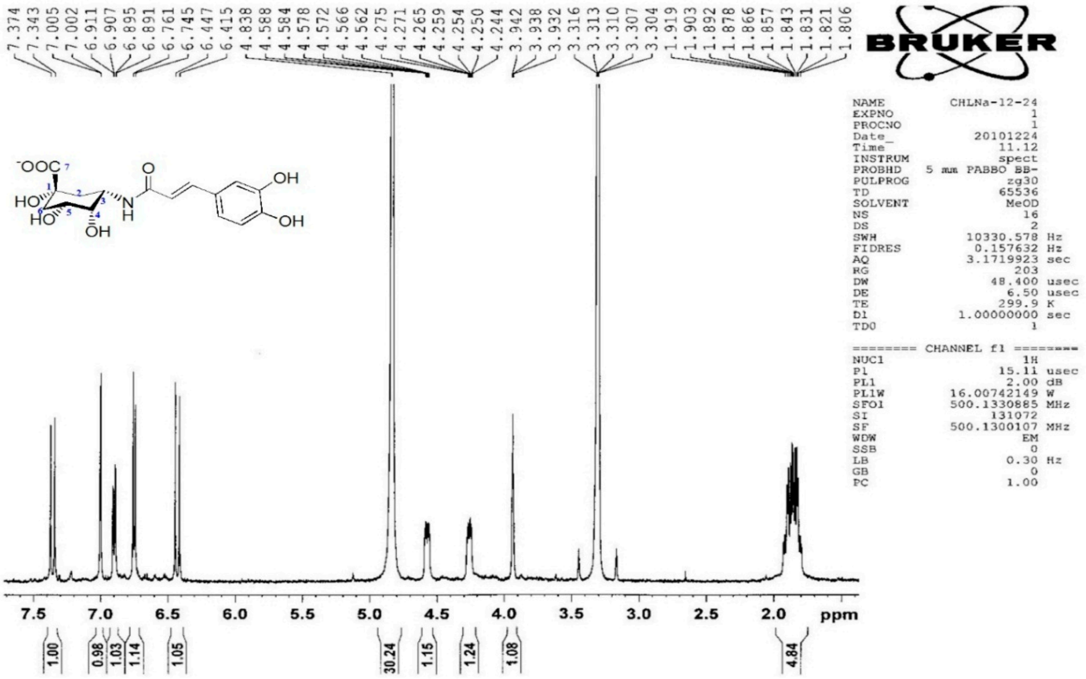

^{13}C-NMR spectrum of **6a**

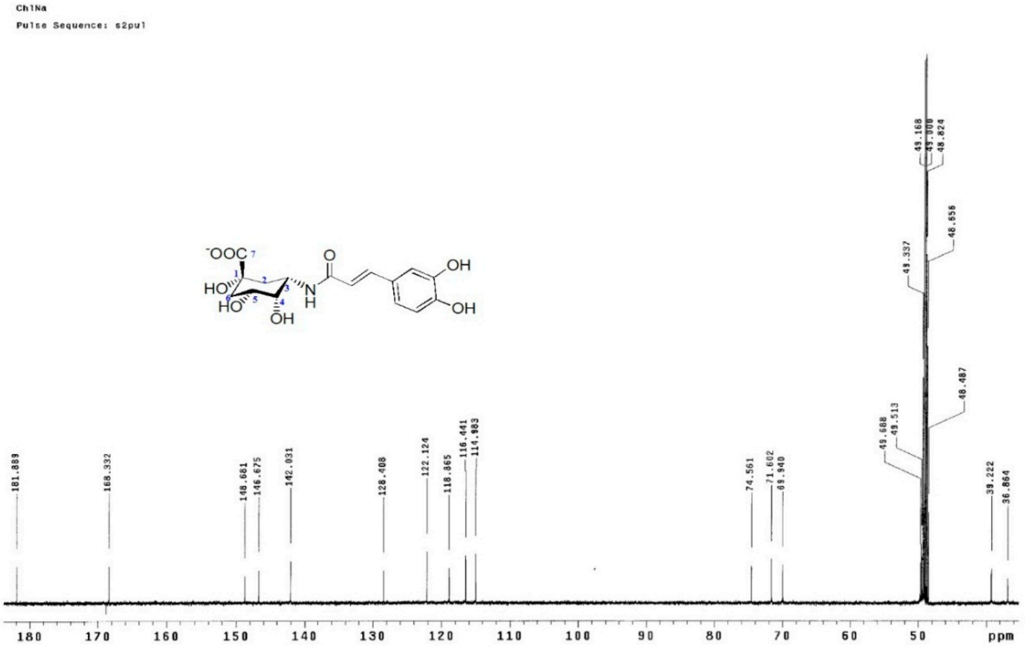

^{1}H-NMR spectrum of **6b**

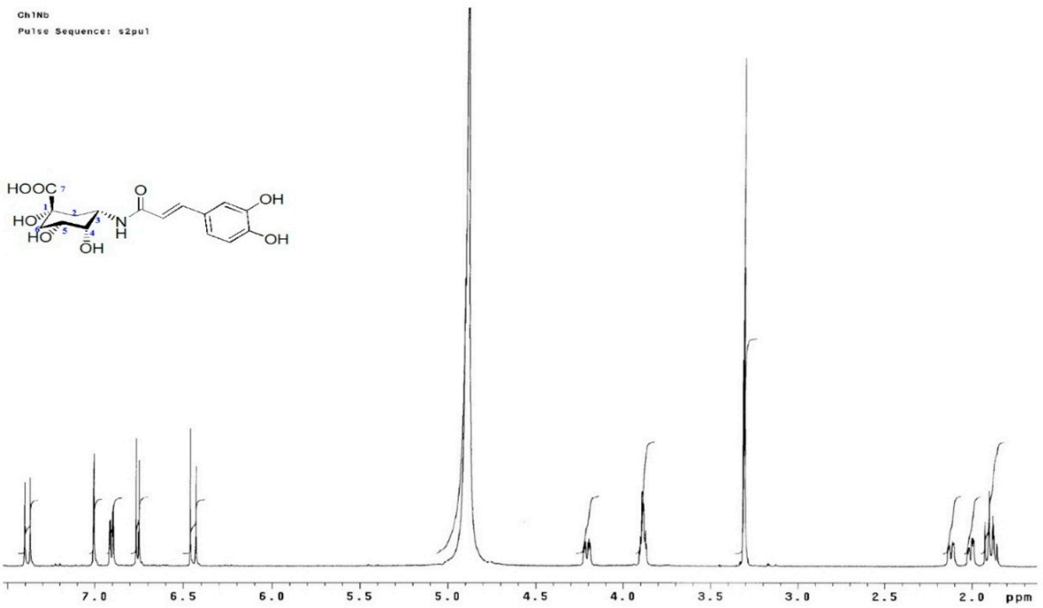

^{13}C-NMR spectrum of **6b**

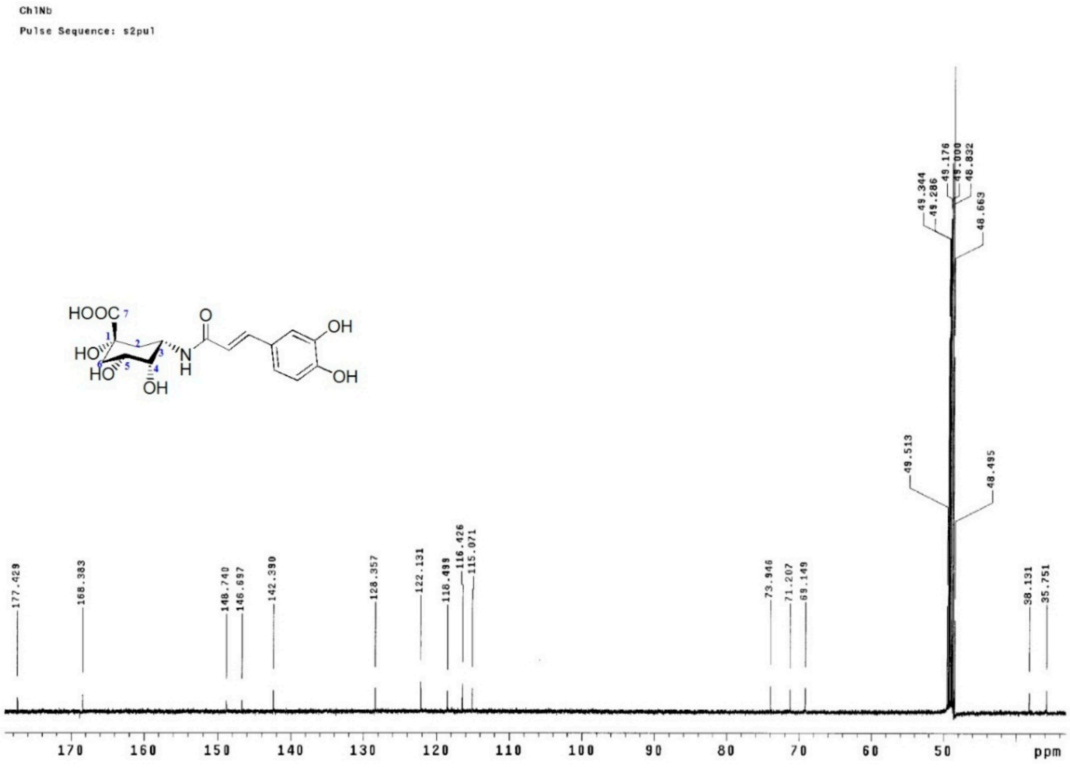

(−) HRFABMS of **6a**

File: ChIN007
Sample: - -
Instrument: AX505W
Inlet: Direct

Date Run: 7-16-2009 (Time Run: 13:16:24)

Ionization mode: FAB-

Scan: 3
Base: m/z 352; 38.8%FS TIC: 3626674

R.T.: .18

#Ions: 65

Selected Isotopes : H $C_{0-16}O_{0-8}N_{0-1}$

Error Limit : 20 mmu

Unsaturation Limits : 0 to 50

Measured Mass	*% Base*	*Formula*	*Calculated Mass*	*Error*	*Unsaturation*
352.10167	100.0%	$C_{16}H_{18}O_8N$	352.10322	-1.6	8.5

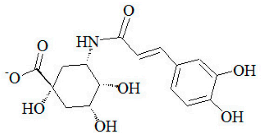

6a $C_{16}H_{18}NO_8^-$
Exact Mass: 352.1032
